# The Effect of the PEEK Cage on the Cervical Lordosis in Patients Undergoing Anterior Cervical Discectomy

**DOI:** 10.3889/oamjms.2015.034

**Published:** 2015-03-21

**Authors:** Salih Gulsen

**Affiliations:** Baskent University Medical Faculty Hospital - Neurosurgery, Maresal Fevzi Cakmak cad. 10. sok. No: 45, Ankara 06540, Turkey

**Keywords:** Cervical, Discectomy, PEEK Cage, Bone Fusion, Osteophyte

## Abstract

**BACKGROUND:**

Loss of cervical lordosis is a significant factor in the development of degeneration of the spine with aging. This degenerative changings of the cervical spine would cause pressure effect on the cervical root and/or medulla spinalis.

**AIM:**

Our goal is to understand the effect of the PEEK cage on cervical lordosis in the early postoperative period. Also, to interpret the effects of one- level, two- level, three-level and four- level disc pathologies on cervical lordosis.

**MATERIAL AND METHODS:**

We retrospectively investigated our archive, and we selected thirty-four patients undergoing anterior cervical discectomy and fusion with PEEK cage filled with demineralized bone matrix (ACDFP).

**RESULTS:**

We determined that ACDFP provides improvement in the cervical lordosis angle in both groups. Also, we found statistically significant difference between group 1 and 2 regarding causes of radiculomyelopathy statistically.

**CONCLUSION:**

We achieved better cervical lordotic angles at the postoperative period by implanting one-level, two-level, three-level or four-level PEEK cage filled with demineralized bone matrix. Also, the causes of cervical root and or medulla spinalis impingement were different in group1 and 2. While extruded cervical disc impingement was the first pathology in group 1, osteophyte formation was the first pathology in group 2.

## Introduction

Neurosurgeons practicing on spinal surgery try to understand the effect of the PEEK cage on cervical lordosis following cervical disc surgery [1-3]. About 60 years ago, Smith and Robinson performed first cervical disc surgery through the anterior approach [4]. In this approach, they placed autograft bone that harvested from patient’s iliac bone into intervertebral disc space to provide the fusion between vertebral bodies. However, about 25 percent of the patients undergoing harvesting autograft for spinal fusion suffered from various complications regarding donor-site [5, 6]. Hence, neurosurgeons start to use Polyether ether ketone (PEEK) cage, titanium cage, artificial disc, allograft bone and bone morphogenic protein to avoid these complications [7-14]. Until now, different authors have already shown the effectiveness of the materials above-mentioned in cervical disc surgery [7-14]. According to these studies, placing PEEK cage, titanium cage or artificial disc to the intervertebral disc space after cervical discectomy provides better cervical lordosis during the postoperative period [7, 9, 10]. As well, the correction of the cervical lordosis would redistribute the vectorial forces on adjacent cervical discs [15-17]. In this way, occurrence of adjacent level degeneration or /and disease- at one level above or below the removed intervertebral disc space would be prevented [15-17]. On the contrary, a few authors asserted that cages and artificial discs would cause kyphosis over time because cages plunge into the corpus of the vertebra cage subsidence [3]. This cage subsidence would cause loss of cervical lordosis and lessened the intervertebral disc height [3,18,19]. In addition to the loss of cervical lordosis and lessened the intervertebral disc height, fusion of the vertebral bodies would prevent the mobility at the site of the fusion [3, 18, 19]. Besides, consequence of this immobility due to fusion, the movement of the lower and upper segments would increase over time [20]. Hence, fusion would result in early disc degeneration at the adjacent cervical segments [20]. As well, if these degenerative discs changing cause radiculopathy and/or myelopathy, its name is adjacent level disease [20]. On the contrary, if there are no signs and symptoms of radiculopathy and/or myelopathy; its name is adjacent level degeneration [20]. We have performed interbody fusion by placing PEEK cage filled with demineralized bone matrix into the intervertebral space following cervical discectomy. In this present study, we collected clinical and radiological data of the patients undergoing anterior cervical discectomy and fusion (ACDF). To explain the effect of the ACDF with PEEK cage, we selected thirty-four consecutive patients undergoing ACDF using by a stand- alone PEEK cage, and we excluded the patients undergoing ACDF with cervical plate. As well, we focused on preoperative and postoperative cervical lordosis angle measurement and its results on clinical signs and symptoms. In addition to cervical lordosis angle, we specifically focused on radiculopathy and myelopathy symptoms in the preoperative period. Also, we separated our patients into two groups. In group 1, fifteen patients undergoing one-level ACDF with PEEK cage. Besides, in group 2, seventeen patients undergoing two-level, one patient undergoing three level and one patient undergoing four-level ACDF with PEEK cage filled with demineralized bone matrix. In the perioperative period, we evaluated cervical lordosis angles, radiculopathy and myelopathy signs and symptoms. We examined each patient at least three times within four months. In addition, we prefer to take control cervical MRI from patients showing no expected surgical results to detect the pathology related adjacent level disease.

## Material and Methods

We retrospectively analysed thirty-four patients undergoing a primary one, two, three and four-level ACDF by the author of this manuscript between June 2011 and June 2014. We included our patients in this study regarding these criteria: Cervical radiculopathy or myelopathy due to cervical disc herniation, bone overgrowth into medulla spinalis and partial ossification of the posterior longitudinal ligament. Besides, we separated our patients into two groups regarding the number of discectomies. We included the patients undergoing one level discectomy to group 1 and patients undergoing two or more level discectomies to group 2. In addition, all patients undergoing ACDF was resistant to conservative measures, including using of neck collar, anti-inflammatory medications and physical therapy. Specifically, any patient with severe arm pain with loss of motor power or myelopathy signs and symptoms, we preferred to ACDF without delaying. Lastly, any patient with systemic disease, such as chronic renal failure, multiple myeloma and history of ischemic stroke had been excluded from the study.

**Figure 1:**
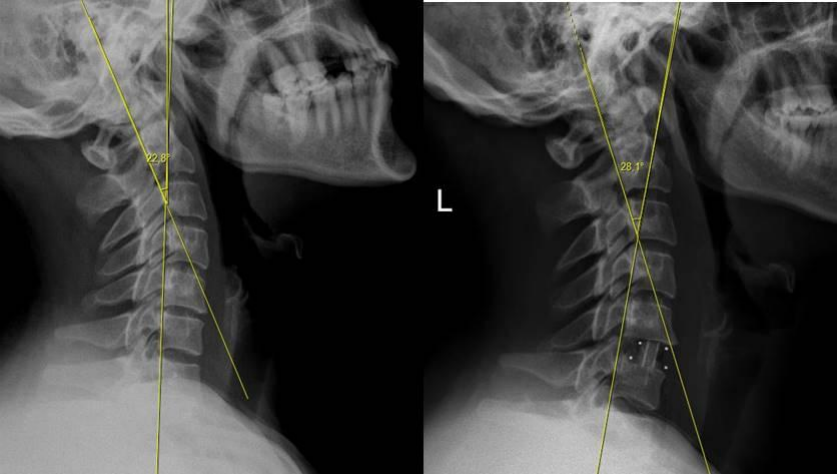
*We measured cervical lordosis angle in degrees on lateral X-ray, and we used Baskent University pacs system (Interpacs Version 1.0.0.0, www.interpacs.com) to measure these angles*.

**Figure 2:**
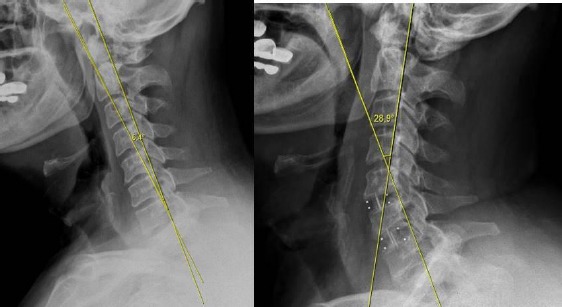
*We measured cervical lordosis angle in degrees on lateral X-ray, and we used Baskent University pacs system (Interpacs Version 1.0.0.0, www.interpacs.com) to measure these angles*.

**Figure 3:**
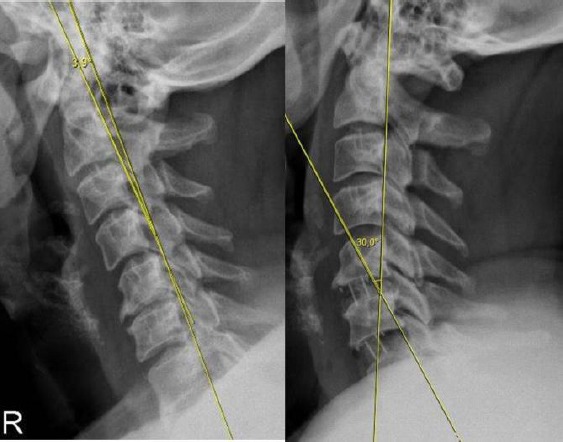
*We measured cervical lordosis angle in degrees on lateral X-ray, and we used Baskent University pacs system (Interpacs Version 1.0.0.0, www.interpacs.com) to measure these angles*.

**Figure 4:**
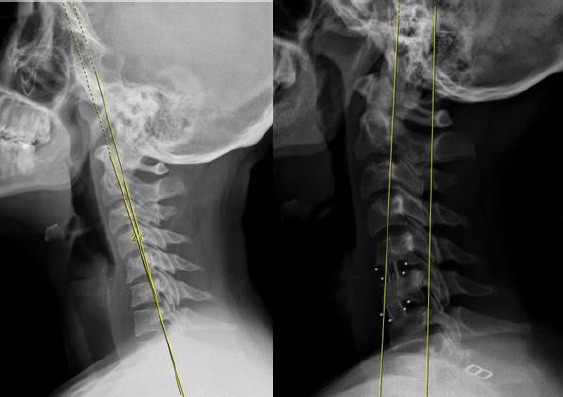
*We measured cervical lordosis angle in degrees on lateral X-ray, and we used Baskent University pacs system (Interpacs Version 1.0.0.0, www.interpacs.com) to measure these angles*.

We used Baskent University pacs system software (Interpacs Version 1.0.0.0, www.interpacs.com) (Fig. 1-4) to measure patients’ cervical lordotic angle with a method previously described by different authors. We used the posterior tangent method. In this method method, an angle of cervical lordosis was measured from C2 through C7 vertebrae on 68 lateral cervical x-rays (Fig. 1-4). (Graphic 1) [21].

### Surgical Technique

We performed the anterior cervical discectomy and fusion (ACDF) is very similar to the procedure described by Robinson [4]. However, we used PEEK cage filled with demineralized bone matrix to provide bone fusion instead of patient’s iliac bone graft. We did microsurgical discectomy (Zeiss neurosurgical microscope) (Oberkochen, Germany) to the level of the uncovertebral joints and the posterior longitudinal ligament. It is noteworthy that we tried to protect the structure of the uncovertebral joint and posterior longitudinal ligament in each case in both groups.

**Figure 5:**
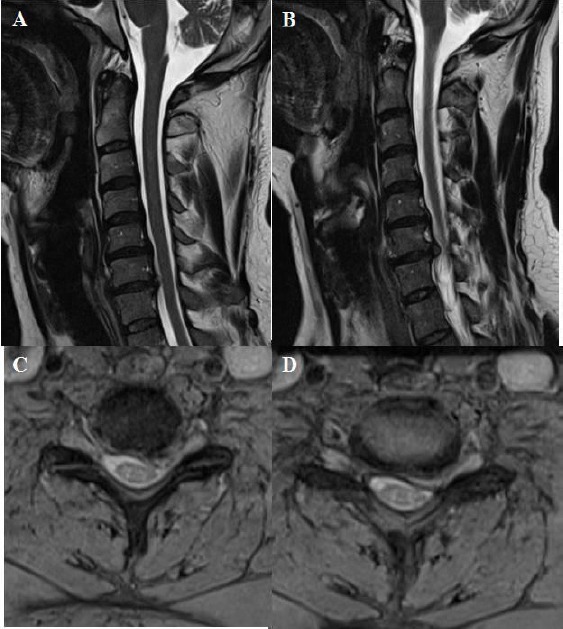
*A, B, C, D: T2 weighted sagittal and axial MRI showing one level cervical root compression due to soft cervical disc extrusion on the left side between cervical 6 and cervical 7. In addition, cervical lordosis angle was measured 22.8 degrees on lateral X-ray at the preoperative period*.

**Figure 6:**
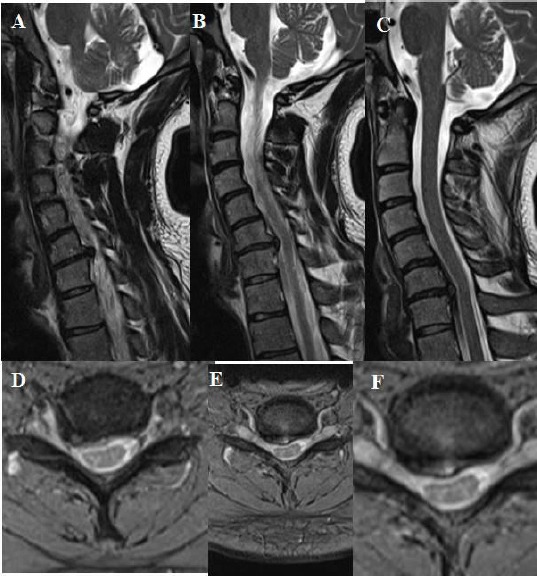
*A, B, C, D, E, F: T2 weighted sagittal and axial MRI showing one level cervical root compression due to soft cervical disc extrusion on the right side between cervical 6 and cervical 7. In addition, sagittal MRI showing cervical kyphosis, and cervical lordosis angle was measured 10 degrees on lateral X-ray at the preoperative period*.

**Figure 7:**
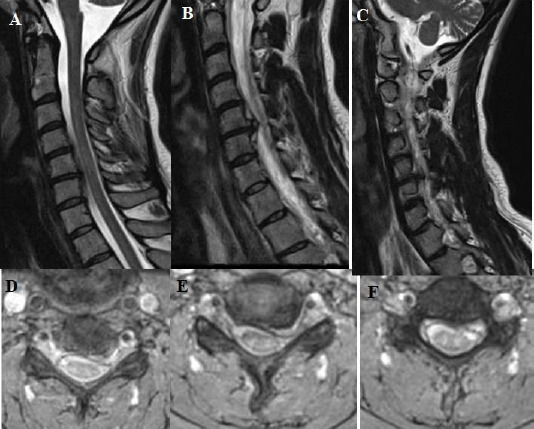
*A, B, C, D, E, F: T2 weighted sagittal and axial MRI showing one level cervical root compression due to soft cervical disc extrusion and minimal osteophyte formation – it is prominent on sagittal images at the 7 B- on the left side between cervical 5 and cervical 6. Besides, cervical lordosis angle was measured 2.4 degrees on lateral X-ray at the preoperative period*.

**Figure 8:**
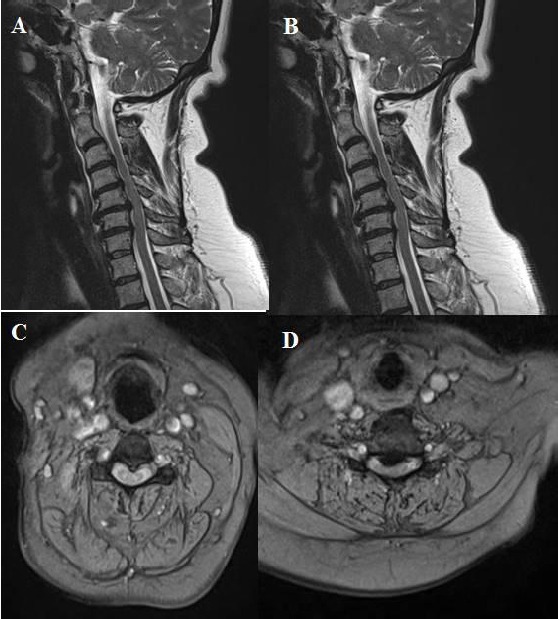
*A, B, C, D: T2 weighted sagittal and axial MRI showing Compression the medulla spinalis at two levels that are between cervical 4 and cervical 5, and between cervical 5 and cervical 6. Osteophyte formation and posterior longitudinal ligament ossification lead to this compression on medulla spinalis. Besides, cervical lordosis angle was measured 0 degree on lateral X-ray at the preoperative period*.

**Figure 9:**
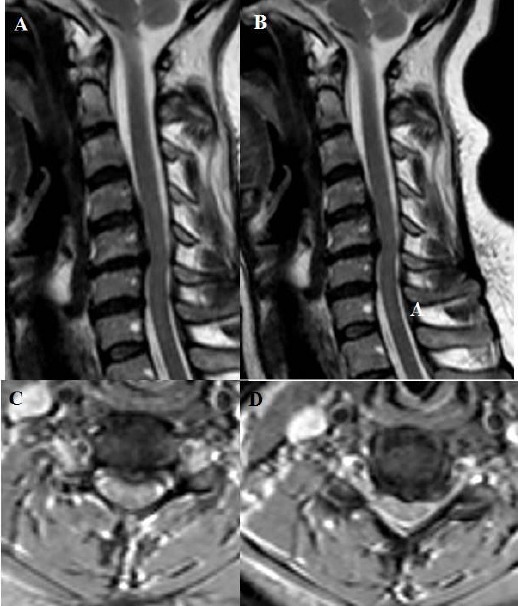
*A, B, C, D: T2 weighted sagittal and axial MRI showing Compression to the medulla spinalis at two levels which are between cervical 3 and cervical 4, and between cervical 4 and cervical 5. Osteophyte formation and posterior longitudinal ligament ossification lead to this compression on medulla spinalis. Besides, cervical lordosis angle was measured 13.6 degrees on lateral X-ray at the preoperative period*.

**Figure 10:**
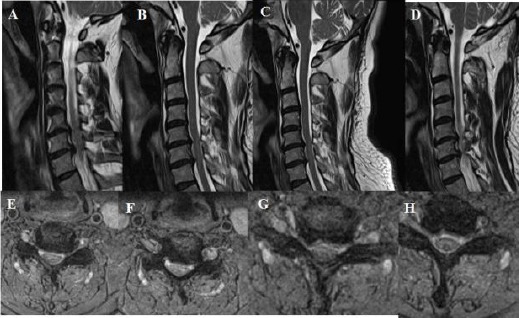
*A, B, C, D, E, F, G, H: T2 weighted sagittal and axial MRI showing Compression to the medulla spinalis at two levels which are between cervical 5 and cervical 6, and between cervical 6 and cervical 7. Osteophyte formation and posterior longitudinal ligament ossification lead to this compression on medulla spinalis. Besides, cervical lordosis angle was measured 6.4 degrees on lateral X-ray at the preoperative period*.

**Figure 11:**
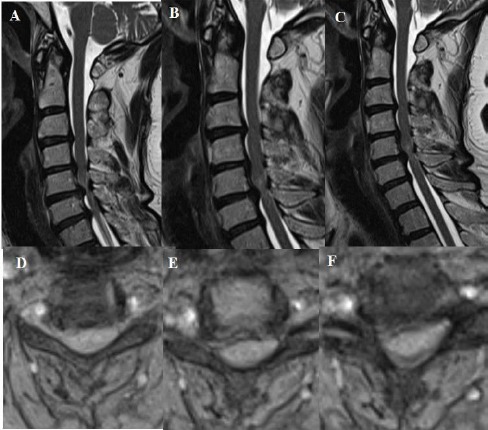
*A, B, C, D, E, F: T2 weighted sagittal and axial MRI showing Compression to the medulla spinalis at four consecutive levels which are from cervical 3 and cervical 4 to cervical 6 and cervical 7. Osteophyte formation and posterior longitudinal ligament ossification lead to this compression on medulla spinalis. Besides, cervical lordosis angle was measured 20.9 degrees on lateral X-ray at the preoperative period*.

However, we could not protect the posterior longitudinal ligament in a few cases because of various reasons. These were tearing of the ligament due to extrude disc fragment, (Fig. 5, 6, 7) partial ossification of the posterior longitudinal ligament (Fig. 8, 9) and indentation of osseous outgrowth into this ligament and medulla spinalis (Fig. 10, 11). Even these situations above mentioned, we managed to keep partially intact to this ligament. For instance, if any osseous outgrowth suppresses the ligament and medulla spinalis at the midline, we prefer to open the ligament in this region instead of to open all parts of the ligament.

Following discectomy, we gently rubbed the bone with a bone curette to provide vascularisation for fusion, and we put the PEEK cage filled with demineralized bone matrix to the intervertebral space. Then, we took lateral X-ray of the cervical spine intraoperatively to determine the placement of the PEEK cage into the intervertebral space. Besides, we took lateral X-ray twenty- four hours after the operation. Next, all patients were instructed to wear a Philadelphia brace for six weeks and a soft collar for an additional two weeks.

### Statistical Methods

We used Student T-test to compare cervical lordosis angles at the preoperative and the postoperative period for each group. In addition, we compared preoperative and postoperative lordosis angle between groups by Student T-test using SPSS 11 (SPSS, Chicago, IL, USA), and P-value below 0.05 was considered significant. In addition to cervical lordosis, we compared the groups regarding

radiculopathy and myelopathy and the osseous overgrowth into medulla spinalis by Chi-square test using SPSS 11 (SPSS, Chicago, IL, USA). Value of p < 0.05 was set to be statistically significant. In addition, we used non- parametric correlation test to demonstrate whether there was any correlation between osteophyte formation and myelopathy signs and symptoms. Value of p < 0.05 was set to be statistically significant correlation (SPSS, Chicago, IL, USA).

## Results

Of the 34 patients, twenty-two patients were male, and twelve patients were female. An average age was 44.23 ± 9.17 years (ranges 26-62 years). We separated our patients into two groups regarding the number of discectomies. We included the patients undergoing one level discectomy to group 1 and patients undergoing two or more level discectomies to group 2.

In group 1, fifteen patients undergoing one-level ACDF. One of the fifteen patients in group 1 undergoing cervical 3-4 ACDF: one of them undergoing cervical 4-5 ACDF, five of them undergoing cervical 5-6 ACDF, and eight of them undergoing cervical 6-7 ACDF. In group 2, one patient undergoing cervical 3-4, 4-5, 5-6, 6-7 ACDF. One patient undergoing cervical 4-5, 5-6, 6-7. Three patients undergoing cervical 3-4, 4-5 ACDF; six patients undergoing cervical 4-5, 5-6 ACDF, and eight patients undergoing cervical 5-6, 6-7 ACDF. We put autologous bone graft harvested from two patients’ iliac bone placed C6-7 intervertebral space. Both of these patients were from group 2, and the first one undergoing cervical 3-4,4-5,5-6,6-7 ACDF and the second one undergoing cervical 4-5,5-6,6-7 ACDF.

While one patient from group 1 had the myelopathy related symptoms and signs, fourteen patients had radiculopathy related symptoms and signs. As well, in group 2, while twelve patients had the myelopathy associated symptoms and signs, seven patients had radiculopathy related symptoms and signs. In the preoperative period, cervical lordosis angle was 11.66 ± 9,17 degrees, on the average, and 16.15 ± 10.47 degrees in the postoperative period in group 1. These values were 15.82 ± 11.54 degrees and 22.07 ± 12.93 degrees respectively in group 2 (Graph.1).

### Statistical Analysis Results

**Figure 12:**
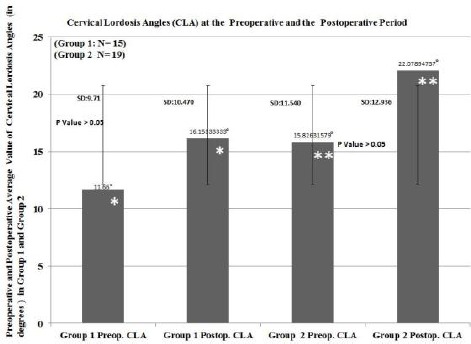
*Student T-test showed no statistically significant difference between preoperative and postoperative cervical lordosis angles in group 1 (P>0.05). P value = 0.221.** Student T-test showed no statistically significant difference between preoperative and postoperative cervical lordosis angles in group 2 (P>0.05). P value = 0.124.

We did not find statistically significant difference between group 1 and group 2 regarding preoperative and postoperative CLA (Fig. 12). However, in both groups postoperative period CLA was higher than that of preoperative period, but it did not show statistical significance (p >0.05), (Fig. 12). More patients had osteophyte formation, which is bone overgrowth into medulla spinalis in group 2 than group 1 (Table 1). It showed statistical significance between group 1 and 2 (p<0.05) (Table 2). Also, Most of the patients in group 2 had myelopathy signs and symptoms, and there was statistical significance between group 1 and 2 (p<0.05), (Table 3, 4). In addition, nonparametric correlation test also showed a significant correlation between osteophyte formation and appearance of myelopathy signs and symptoms in group 2 (Table 5).

**Table 1:** Cross tabulation analysis of cases in both groups regarding with osteophyte at the intervertebral disc space.

			OSTEOFITFORMATIONATHELE VELOFOISCECTOMY		Total

			There is osteophyte formation	There is no osteophyte formation	
Groups	Group 1	Count	1	14	15
		Expected Count	75	7.5	15.0
		% within Groups	6.7%	93.3%	100.0%
		% within OSTEOFITFORMATIONATHELEVELOFDISCECT OMY	5.9%	82.4%	44.1%
	
	Group 2	Count	16	3	19
		Expected count	95	9.5	19.0
		% within Groups	84.2%	15.8%	100.0%
		% within OSTEOFITFORMATIONATHELEVELOFDISCECT OMY	941%	17.6%	55.9%

Total		Count	17	17	34
		Expected Count	17.0	17.0	34.0
		% within Groups	50.0%	50.0%	100.0%
		% within OSTEOFITFORMATIONATHELEVELOFDISCECT OMY	100.0%	100.0%	100.0%

**Table 2:** Chi-Square test results regarding with osteophyte formation.

	Value	df	Asymp. Sig. (2-Sided)	Exact Sig. (2- Sided)	Exact Slg. (1- Sided)
Pearson Chi-Square	20.161^a^	1	.000		
Continuity Correction^b^	17.179	1	.000		
Likelihood Ratio	23.212	1	.000		
Fisher's Exact Test				.000	.000
Linear-by-Linear Association	19.568	1	.000		
N of Valid Cases	34				

a. 0 cells (.0%) have expected count less than 5. The minimum expected count is 7.50.

b. Computed only for a 2x2 table

**Table 3:** Cross tabulation analysis of cases in both groups regarding with myelopathy and radiculopathy.

			Radiculomyelopathy		Total

			Radiculopathy signs and symptoms	Myelopathy signs and symptoms	
Groups	Group 1	Count	14	1	15
		Expected Count	9.3	5.7	15.0
		% within Groups	93.3%	6.7%	100.0%
		% within Radiculomyelopathy	66.7%	7.7%	44.1%
	
	Group 2	Count	7	12	19
		Expected Count	11.7	7.3	19.0
		% within Groups	36.8%	63.2%	100.0%
		% within Radiculomyelopathy	33.3%	92.3%	55.9%

Total		Count	21	13	34
		Expected Count	21.0	13.0	34.0
		% within Groups	61.8%	38.2%	100.0%
		% within Radiculomyelopathy	100.0%	100.0%	100.0%

**Table 4:** Chi-Square test results regarding with myelopathy and radiculopathy.

	Value	df	Asymp. Sig. (2-sided)	Exact Sig. (2- sided)	Exact Sig. (1- sided)
Pearson Chi-Square	11.327^a^	1	.001		
Continuity Correction^b^	9.061	1	.003		
Likelihood Ratio	12.878	1	.000		
Fisher's Exact Test				.001	.001
Linear-by-Linear Association	10.994	1	.001		
N of Valid Cases	34				

a. 0 cells (.0%) have expected count less than 5. The minimum expected count is 5.74.

b. Computed only for a 2x2 table

**Table 5:** Non- parametric correlation analysis showing the correlation between osteophyte formation and myelopathy signs and symptoms.

		Value	Asymp. Std. Error^a^	Approx. T^b^	Approx. Sig.
Interval by Interval	Pearson's R	.577	.123	3.998	.000^c^
Ordinal by Ordinal	Spearman Correlation	.577	.123	3.998	.000^c^
N of valld Cases		34			

a. Not assuming the null hypothesis.

b. Using the asymptotic standard error assuming the null hypothesis.

c. Based on normal approximation.

## Discussion

In our presented study, we performed seventy-one cervical discectomies from cervical 3-4 to cervical 6-7 to 34 patients according to the description by Robinson and Smith [4]. About for sixty years, neurosurgeons have used anterior cervical discectomy with fusion (ACDF) to treat cervical spondylosis disease [4, 22]. These include posterior or posterolateral disc herniation, bulging of the ligamentum flavum, osteophyte formation, and ossification of the posterior longitudinal ligament [7, 8, 10, 22-24]. Anterior cervical discectomy with fusion has been evolved over time.

For example, neurosurgeons prefer to use allograft bone or PEEK cage filled with demineralized bone matrix to provide fusion and cervical lordosis [13, 14, 25]. In addition, Titanium made artificial cervical discs and Titanium cages coated bone morphogenic protein has been used for different purposes [11-13, 24-26]. Specifically, artificial disc materials are tamped into the level of discectomy to prevent adjacent level degeneration [10]. That is the problem following every kind of fusion of the cervical spine [20]. On the other hand, performimg multiple level discectomies in cervical spondylosis may cause more problems than one level discectomy because patients undergoing multilevel discectomy may suffer from cervical mobility at the levels of the cervical discectomies [20, 27, 28]. As well, this mobility may cause either kyphosis or pseudoarticulation or both of them [20, 27, 28]. For this reason, neurosurgeons prefer to fix intervertebral place with cervical plate following placement of the cage filled with osteoinductive bone substitute to prevent these complications [20, 23, 25, 28].

Anterior cervical plate would provide immobilization at the level of discectomy for bone fusion, but it may cause more hypermobility at the adjacent cervical disc regions than stand-alone cages [25, 27, 29]. Besides, It would cause adjacent level disease requiring secondary cervical disc surgery[25,27,29]. As well, cage subsidence would cause two problems [16-19]. Firstly, narrowing of disc space because of the cage subsidence would pressure on cervical root just above the pedicle of the cervical vertebra [16-20].

Secondly, cage subsidence would cause loading anterior part of the vertebra corpus, and its vectorial force would induce cervical lordosis to hypolordosis or kyphosis [16-20, 30]. As well, hypolordosis accelerates the degenerative process [20, 30, 31]. Besides, one-level cervical discectomy is complicated surgery because following one level discectomy of C5 to C6 or C6 to C7 with stand–alone cage would cause adjacent segment degeneration due to the inherent mobility of these segments [20, 31]. However, whether this degeneration may result in normal ageing process or the effect of the ACDF is a controversial issue [17-20, 30-33]. Recent publications have advocated that this degeneration may occur in the typical progression of cervical spondylosis rather than being a direct complication of cervical fusion [33-34]. Nevertheless, the success rate of ACDF is satisfactory regarding long-term follow-up studies [22, 23]. Namely, the rate of adjacent level disease is about 3 % for the first ten years following an ACDF [22, 23].

Development of the adjacent level disease depends on few factors following ACDF [20, 22, 23]. These are the presence of pre-existing degenerative changing at the adjacent levels, fusions ending adjacent to C5-C6 and C6-C7, and unnoticed degenerative changing of the adjacent level in the preoperative period [20, 27, 34]. Besides, a few authors have also emphasized the importance of keeping sagittal alignment in lordotic state, and they advocate that changing of the lordotic angles accelerate the degenerative process [16, 27, 29, 30]. For this reason, along with ACDF with PEEK cage, neurosurgeons try to keep intact the posterior longitudinal ligament to prevent cervical spinal stability and sagittal alignment [20, 22, 23]. However, neurosurgeons cut posterior longitudinal ligament if it is necessary to reach below this ligament for extracting the extrude disc, osteophyte and ossification of the ligament [20, 23]. Similarly, surgical procedures would promote spondylotic degeneration and distort the cervical lordosis angle [20, 27, 34]. These methods include extensive dissection of longus colli muscles on both sides of the anterior part of the vertebra body, extensive curreting of the uncovertebral joint and also extensive curreting of the vertebral end plates [16, 25, 27, 32, 34]. Finally, all of them would reproduce adverse effects on naturally occurring degenerative process [16, 25, 27, 32, 34]. In addition, extensive bone curettage would cause PEEK cage subsidence because subcortical bone has not ability to resist loading force as to the cortical layer of the corpus [16, 20, 32, 34].

On the other hand, in cervical spondylosis cases, authors perform posterior, anterior or combined approach with different modalities [35-39]. For example, a few authors perform cervical artificial disc replacement following anterior cervical discectomy instead of performing fusion to prevent adjacent segment dejeneration [19]. The others perform ACDF with Titanium box cage with bone morphogenic protein to provide fusion [13]. The other authors prefer posterior approach with its different modalities, including cervical laminectomy, cervical lamimoplasty, fixation with pedicule screws or lateral mass screws and cervical discectomy using posterior approaches [35-39].

It is important to choice appropriate surgical approach — anterior or posterior— decompression and reconstruction method because of the multitude of the surgical techniques and different materials for reconstruction and fusion [39].

Generally, if any pathology impinges on the medulla spinalis from the anterior side, including osteophyte, segmental ossification of PLL, neurosurgeons perform anterior approach. However, in cases with kyphosis and hypolordosis, if the pathology impinges on the posterior surface of the medulla spinalis, surgery through posterior way would cause increasing the kyphosis and turning into hypolordosis to kyphosis. In addition, medulla spinalis would not move to the posteriorly due to kyphotic and hypolordotic angles, and patients’ myelopathy would deteriortae because of increased kyphotic angle [35, 36, 39]. In this situation, neurosurgeons may choice combined approach to prevent kyphosis and hypolordosis [37, 39].

In our series, we performed ACDF with PEEK cage filled with demineralized bone matrix to the patients with from one level to four level without applying anterior plate for fixation. Our detailed results were presented at the Fig. 12 and Table 1-5. Also, we presented a few patients preoperative and postoperative cervical lordosis angle.

Regarding our study, we try to keep anatomical structure to protect cervical spinal stability and sagittal alignment. It is noteworthy that we selected our patients with a few factors for ACDF procedure. Firstly, during the examination, we focused on radiculopathy and myelopathy -motor power, absence or presence of pathologic reflexes, clonus the quality of the deep tendon reflexes and intensity of the pain of the patient. Then, we decided to take cervical magnetic resonance imaging (MRI) and lateral and anteroposterior X-ray images. If there was concordance with the signs and symptoms of the patient with their MRI scans and X-ray, we decided to operate the patient. Besides, if there was preexisting degenerative changing adjacent level of the C5-C6 or C6-C7, we preferred to perform two level discectomy rather than one level discectomy to prevent the development of adjacent level disease. Patients undergoing one- level ACDF in group 1, and patients undergoing two- level, three-level and four- level ACDF in group 2. Patients’ cervical lordotic angles were hypolordotic in both groups except that one patient from group 2. This patient’s preoperative CLA was 49°, and postoperative CLA was 44.2°.

If the cervical lordotic angle falls within the range of 30°-45°, it is accepted within the normal range [21]. Thirty-three patients of the thirty four patients had hypolordosis in the preoperative and postoperative period. However, one patient from group 2 had hyperlordosis in the preoperative period and normal cervical lordotic angle in the postoperative period.

While cervical lordotic angle, on the average, at the preoperative and postoperative period in group 1 were 11.66° and 16.15°, and correspondingly 5.82° and 22.07 in group 2. In group 1, insertion of PEEK with cage after one level discectomy increased the cervical lordotic angle, on the average, 4.49°. While in group 2, insertion of two PEEK cage after two, three or four level discectomy increased the cervical lordotic angle, on the average, 6.25°. In both groups, student-T-test showed no statistically significant difference between preoperative and postoperative period regarding cervical lordotic angle (P>0.05). Other than cervical lordotic angle, we investigated the effect of osteophyte formation, ossification of posterior longitudinal ligament and extruded disc with myelopathy and radiculopathy in group 1 ad group 2. Chi-square test showed a significant difference between groups 1 and group 2 regarding myelopathy, radiculopathy, osteophyte formation, ossification of posterior longitudinal ligament and extruded disc. While one of the fifteen patients in group 1 had osteophyte, in group 2 sixteen of nineteen patients had osteophyte or posterior longitudinal ligament ossification (PLLO). This degenerative changing would hinder expected improvement of cervical lordosis because of stiffness in cervical spinal region due to PLLO and osteophyte formation. However, this reasoning is not enough to explain why the patients from group 1 showed no better improvement regarding CLA. There would be two reason for this effect. Firstly, during conservative treatment period, using cervical collar and application of intermittent cervical traction would provide comfortable time for the patients. While these methods provide the widening of the vertebral foramen that is the exit point of cervical roots, on the other hand, they would induce hypolordosis and accelerate the degeneration of cervical vertebral discs. Additionally, neck muscle spasm would contribute this adverse effect on the sagittal alignment in the period of neck and arm pain during cervical disc degeneration [20]. As well, decreasing height of the intervertebral disc causes osseous pressure on cervical roots [1, 30, 32]. Then, irritation of cervical nerve root causes pain, and pain leads to straightening of the neck as a compensation mechanism to lessen the pressure on the nerve root[1,30,32]. Namely, straightening of cervical lordosis enlarges the foramen that is the exit point of the cervical nerve root [1, 20, 30, 32]. However, this compensation is not endless process, when lordosis turns into kyphosis foramen start to narrow instead of enlarging [1, 30, 32]. Over time, those patients who have had hypolordotic cervical spine would develop PLLO or osteophyte formation or extrusion of the intervertebral disc into posterior or posterolateral part of the foramen. Initially, one or two or three of these pathologic process occur, and patients suffer from myelopathy or radiculopathy or both of them [1, 30, 32]. Neurosurgeons Performing ACDF would remove extrude disc or PLLO or osteophyte, but they would not wipe out degenerative and/or inflammatory process of the cervical spine. Implanting one-level, two-level, three-level or four-level PEEK cage filled demineralized bone matrix may add a positive impact on the cervical lordotic angle, but this improvement may not provide ideal cervical lordotic angles. Besides, it would not stop the aging process or degeneration process, but it provide better life quality to the patients suffering from cervical spondylosis. ACDF is still the best solution in suitably selected patients.
